# Molten salt electrosynthesis of Cr_2_GeC nanoparticles as anode materials for lithium-ion batteries

**DOI:** 10.3389/fchem.2023.1143202

**Published:** 2023-02-17

**Authors:** Zhongya Pang, Feng Tian, Xiaolu Xiong, Jinjian Li, Xueqiang Zhang, Shun Chen, Fei Wang, Guangshi Li, Shujuan Wang, Xing Yu, Qian Xu, Xionggang Lu, Xingli Zou

**Affiliations:** ^1^ State Key Laboratory of Advanced Special Steel and Shanghai Key Laboratory of Advanced Ferrometallurgy and School of Materials Science and Engineering, Shanghai University, Shanghai, China; ^2^ Center for Hydrogen Metallurgy Technology, Shanghai University, Shanghai, China; ^3^ Shanghai Institute of Applied Physics, Chinese Academy of Sciences, Shanghai, China; ^4^ School of Materials Science, Shanghai Dianji University, Shanghai, China

**Keywords:** MAX phase, Cr_2_GeC, molten salt electrosynthesis, lithium-ion batteries, energy storage

## Abstract

The two-dimensional MAX phases with compositional diversity are promising functional materials for electrochemical energy storage. Herein, we report the facile preparation of the Cr_2_GeC MAX phase from oxides/C precursors by the molten salt electrolysis method at a moderate temperature of 700°C. The electrosynthesis mechanism has been systematically investigated, and the results show that the synthesis of the Cr_2_GeC MAX phase involves electro-separation and *in situ* alloying processes. The as-prepared Cr_2_GeC MAX phase with a typical layered structure shows the uniform morphology of nanoparticles. As a proof of concept, Cr_2_GeC nanoparticles are investigated as anode materials for lithium-ion batteries, which deliver a good capacity of 177.4 mAh g^−1^ at 0.2 C and excellent cycling performance. The lithium-storage mechanism of the Cr_2_GeC MAX phase has been discussed based on density functional theory (DFT) calculations. This study may provide important support and complement to the tailored electrosynthesis of MAX phases toward high-performance energy storage applications.

## Highlights

• Cr_2_GeC nanoparticles were first prepared from oxides/C precursors by molten salt electrolysis.
• The electrosynthesis mechanism of Cr_2_GeC involving electro-separation and *in situ* alloying processes has been investigated systematically.
• The as-prepared Cr_2_GeC nanoparticles as anode materials for lithium-ion batteries deliver an impressive capacity of up to 177.4 mAh g^−1^ at 0.2 C.

## 1 Introduction

The MAX phases are ternary lamellar-structure transition metal carbides and/or nitrides with a general formula of M_n+1_AX_n_, where M is an early transition metal; A is an A-group element; X is C and/or N; and n is 1, 2, or 3 (Barsoum, 2000; Sokol et al., 2019; Fatima et al., 2020). In recent years, MAX phases have received widespread attention due to their superior physical and chemical properties, such as excellent thermal/electrical conductivity (Jin et al., 2020), thermal-shock resistance (Li et al., 2014), high-temperature oxidation resistance (Drouelle et al., 2020), and mechanical properties (Tan et al., 2021). The bulk MAX phases were commonly synthesized by hot pressing and spark plasma sintering (Ghasali et al., 2021; Zhang et al., 2021). The molten salt electrolysis method as a simple and economical strategy has broad appeal for the fabrication of MAX phase powders. Oxides or even multi-component ores and graphite powders can be used as raw materials to synthesize MAX phase powders by molten salt electrolysis. Molten salt as an ionic solvent facilitates the mass transfer and nucleation/growth processes, resulting in finer and more homogeneous particle products (Liu et al., 2013; Li et al., 2021; Li et al., 2022). Up until now, some MAX phases (V_2_AlC, Ti_3_AlC_2_, V_4_AlC_3_, Cr_2_AlC, *etc.*) have been synthesized using the molten salt electrolysis method (Amr, 2016; Liu et al., 2020; Pang et al., 2020; Gao et al., 2022).

Lithium-ion batteries (LIBs) are one of the most widely used electrochemical energy storage devices due to the advantages of high energy density, high Coulombic efficiency, and long service life (Kim et al., 2019; Zhao et al., 2020)*.* Energy storage materials have been continuously investigated to support the development of high-performance LIBs. MAX phases with special laminated structures and excellent metal conductivities have been considered as potential lithium-storage hosts (Xu et al., 2016; Chen et al., 2018; Luan et al., 2019; Zhao et al., 2019). Xu et al. investigated the reversible electrochemical intercalation behavior of Li ions in Ti_2_SC and Ti_3_SiC_2_ MAX phases and concluded that particle size has an important influence on the electrochemical properties of MAX phases. The nanoscale Ti_2_SC delivered the initial reversible capacity of about 80 mAh g^−1^ (at 4 C), which increases to about 180 mAh g^−1^ after 1,000 cycles (Xu et al., 2016). Chen et al. confirmed that partially etched Ti_3_AlC_2_ has potential as an anode for high-capacity LIBs through the alloying of Al with Li (Chen et al., 2018). Li et al. prepared the V_2_SnC MAX phase with a high weight capacity of 490 mAh g^−1^ (volume capacity of 570 mAh cm^−3^) *via* the molten salt method, and a charge storage mechanism involving dual redox reactions of V_2_C–Li and Sn–Li was proposed (Li et al., 2021). In general, MAX phases have attracted increasing attention for applications as Li-storage anodes.

Ge, with an excellent Li ion diffusion rate and high electrical conductivity, has been considered a promising anode material candidate for LIBs (Hu et al., 2016). However, the volume expansion (about ∼250% for Li_15_Ge_4_) of Ge during the Li insertion/extraction process severely hampers its energy storage properties. Cr_2_GeC is one of the MAX phases, and the A-layer atom is Ge. The stable Cr_2_GeC MAX phase is expected to take advantage of the two-dimensional structural properties of the MAX phase and the Li-storage property of metallic Ge. If the typical alloying mechanism of Li_15_Ge_4_ in the Cr_2_GeC MAX phase is considered as the basis, the theoretical capacity can reach 535 mAh g^–1^ (Xu et al., 2016). In this work, Cr_2_GeC nanoparticles were easily prepared by molten salt electrolysis of oxides/C precursors and evaluated as anode materials for LIBs for the first time. The results show that the as-prepared Cr_2_GeC with a refined particle size delivers a high rate and excellent cycling performance, exhibiting an attractive Li storage capacity.

## 2 Materials and methods

### 2.1 Molten salt electrosynthesis of Cr_2_GeC

Commercial Cr_2_O_3_ (3 μm, 99.5%, Sinopharm Chemical Reagent Co., Ltd.), GeO_2_ (500 nm, 99.9%, Sinopharm Chemical Reagent Co., Ltd.), graphite powders (10 nm, 99.8%, Sinopharm Chemical Reagent Co., Ltd.) with different molar ratios (1:1:1, 1:2:1, 1:3:1, and 1:4:1), and 10 wt% polyvinyl butyral (PVB, Sinopharm Chemical Reagent Co., Ltd.) were mixed by ball-milling at 300 r/min for 5 h to prepare the powdered Cr_2_O_3_/GeO_2_/C precursor. About 0.5 g of the obtained mixed powders were pressed under 10 MPa to fabricate a Cr_2_O_3_/GeO_2_/C disc (10 mm in diameter). The Cr_2_O_3_/GeO_2_/C disc was wrapped by nickel foam and fixed on a Mo wire (2 mm in diameter, Shanghai Non-Ferrous Metals (Group) Co., Ltd.) to form the Cr_2_O_3_/GeO_2_/C cathode system. A high-purity graphite rod (5 mm in diameter, 99.999%, Shanghai Carbon Co., Ltd.) fixed with the Mo wire was used as the anode. CaCl_2_ and NaCl (Shanghai Aladdin Biochemical Technology Co., Ltd.) were commonly baked at 300–400°C for 24–48 h and then used as electrolytes in a 1:1 M ratio. The electrodes and mixed salts were assembled in a corundum crucible to form an electrolytic cell, which was then placed in an electrolysis furnace sealed on one end. High-purity Ar gas was continuously introduced into the electrolytic furnace to create an inert atmosphere. The electrolysis furnace temperature was then ramped up to 700°C with a heating rate of 5°C/min. Pre-electrolysis was then performed between two graphite rods (5 mm in diameter, 99.999%, Shanghai Carbon Co., Ltd.) at 2.0 V for 2–5 h to eliminate residual purities in molten salts. A constant voltage of 3.0 V was applied between the Cr_2_O_3_/GeO_2_/C cathode and the graphite anode for pre-set times. After electrolysis, the obtained electrolytic samples were washed with deionized water to remove solid salts and then, dried at 100°C in a vacuum drying oven for further characterization.

### 2.2 Lithium-storage performance tests of Cr_2_GeC

The two-electrode CR2032-type coin cell was fabricated to evaluate the lithium-storage performance of the as-prepared Cr_2_GeC. In detail, a slurry made by mixing 80 wt% Cr_2_GeC as active materials, 10 wt% acetylene black (Taiyuan Lizhiyuan Technology Co., Ltd.), and 10 wt% polyvinylidene fluoride (PVDF, Taiyuan Lizhiyuan Technology Co., Ltd.) in N-methyl pyrrolidone (NMP, Taiyuan Lizhiyuan Technology Co., Ltd.) was coated on a Cu foil; then, the obtained Cu foil coated with the slurry was dried under vacuum at 80°C for 10 h. The disc-shaped electrodes (12 mm in diameter) were cut off from the dried Cu foil. In addition, the lithium metal foil, 1.0 M LiPF_6_, and the polypropylene membrane (Taiyuan Lizhiyuan Technology Co., Ltd.) were used as the counter electrode, electrolyte, and separator, respectively. The coin cells were assembled in an argon glovebox.

### 2.3 Material characterization

The phase composition of the samples was analyzed by X-ray diffraction (XRD, Bruker D8 Advance). The morphology, microstructure, and elemental distribution of the samples were characterized by scanning electron microscopy (SEM, JEOL JSM-6700F), transmission electron microscopy (TEM, JEM-2100F), and the affiliated energy dispersive X-ray spectrometer (EDS). A Bio-Logic HCP-803 electrochemical workstation was used to record the current–time curve of the electrolysis process and cyclic voltammetry curves of the fabricated coin cell. The charge–discharge tests of coin cells were carried out on a NEWARE CT-4000 battery test system. For computational details, all density functional theory (DFT) calculations were performed using the Vienna Ab-initio Simulation Package (VASP). The generalized gradient approximation (GGA) developed by Perdew, Burke, and Ernzerhof (PBE) was used as the exchange-correlation potential. The cutoff energy is set to 400 eV. A Monkhorst–Pack grid of 6 × 6 × 6 was used for bulk lattice optimization, and a Monkhorst–Pack grid of 3 × 3 × 1 was used for slabs. Electronic and ionic optimizations were performed using a self-consistent field (SCF) energy criterion of 10^–4^ eV and a maximum force of 0.001 eV/Å. The 2 × 2 × 1 supercell of the Cr_2_GeC (010) slab contains 38 atoms, where the bottom three atomic layers were fixed. A vacuum layer of 15 Å was used to prevent the interaction between the near slabs.

## 3 Results and discussion

**FIGURE 1:**
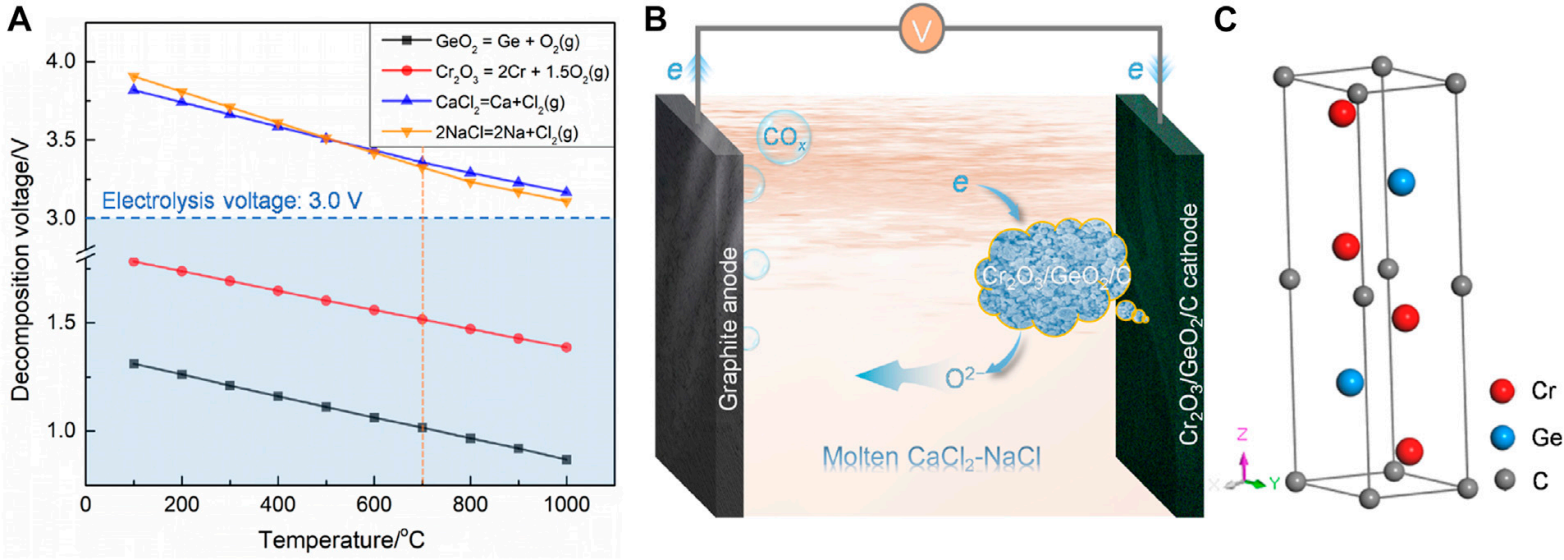
**(A)** Temperature-dependent theoretical decomposition voltages of Cr_2_O_3_, GeO_2_, CaCl_2_, and NaCl. **(B)** Schematic diagram of the electrolysis process. **(C)** Crystal structure of Cr_2_GeC.

Cr_2_O_3_/GeO_2_/C used as a cathode was directly electrolyzed to prepare Cr_2_GeC in molten CaCl_2_–NaCl. Theoretical analyses of Cr_2_O_3_, GeO_2_, CaCl_2_, and NaCl were first performed based on Gibbs free energy, and the calculated temperature-dependent decomposition voltage plots are shown in Figure 1A. The results show that the applied voltage of 3.0 V is sufficient to electrochemically separate the oxygen from the oxides (Cr_2_O_3_ and GeO_2_) in the cathode at a wide temperature range below 1,000°C, in the case of avoiding the decomposition of chloride molten salts. Theoretically, oxygen ions ionized from cathodic Cr_2_O_3_ and GeO_2_ and discharged at the anode at the applied voltage. The *in situ* alloying reaction between the electrochemically reduced metals Cr, Ge, and C was expected to induce the formation of Cr_2_GeC. Figures 1B and C show the schematic of this electrolysis process and the crystal structure of the Cr_2_GeC MAX phase.

**FIGURE 2:**
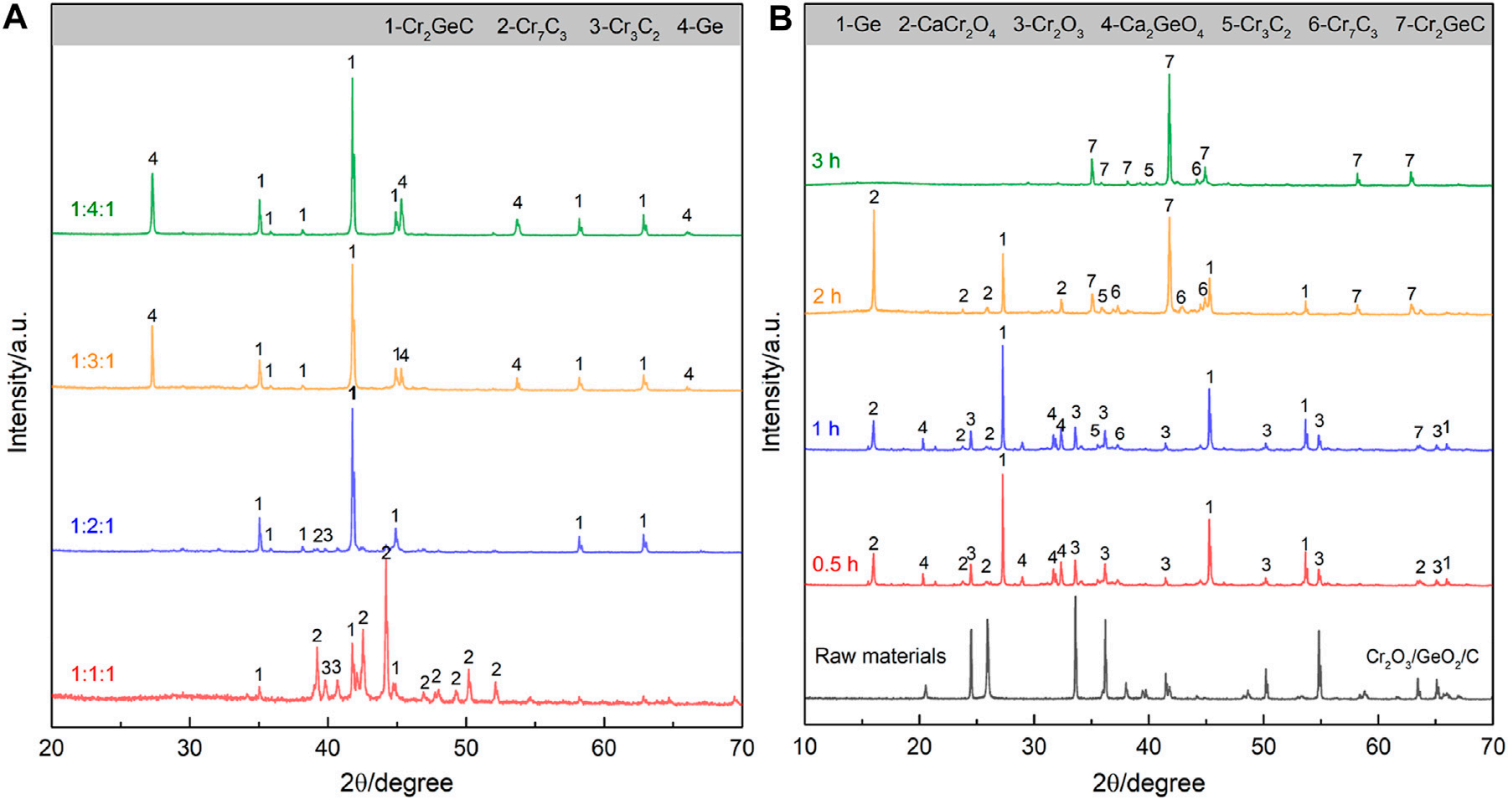
**(A)** XRD patterns of the samples obtained by the electrolysis of Cr_2_O_3_/GeO_2_/C with different molar ratios (1:1:1, 1:2:1, 1:3:1, and 1:4:1) at 700°C for 3 h. **(B)** XRD patterns of the samples obtained by the electrolysis of Cr_2_O_3_/GeO_2_/C (with a molar ratio of 1:2:1) at 700°C for different times.

After the aforementioned theoretical analysis, the electrosynthesis of the target Cr_2_GeC was first attempted using Cr_2_O_3_/GeO_2_/C with a molar ratio of 1:1:1 in molten CaCl_2_–NaCl at a relatively moderate temperature of 700°C. However, the XRD result (Figure 2A) shows that the electrolytic sample contains mixed phases of Cr_2_GeC, Cr_7_C_3_, and Cr_3_C_2_, indicating a significant loss of the Ge content. Therefore, excess GeO_2_ was further supplied into the cathode to compensate for the loss of Ge. The samples obtained by the electrolysis of Cr_2_O_3_/GeO_2_/C precursors with different molar ratios of 1:2:1, 1:3:1, and 1:4:1 were further analyzed by XRD. It can be confirmed that Cr_2_GeC can be synthesized from Cr_2_O_3_/GeO_2_/C with a molar ratio of 1:2:1, while a further increase in the GeO_2_ content (i.e., cases of 1:3:1 and 1:4:1) results in the generation of metallic Ge as the second phase in Cr_2_GeC. On this basis, the electrolysis of Cr_2_O_3_/GeO_2_/C with a molar ratio of 1:2:1 was further analyzed to understand the formation of the Cr_2_GeC MAX phase. Figure 2B shows XRD patterns of the samples obtained through the electrolysis of the Cr_2_O_3_/GeO_2_/C cathode (with a molar ratio of 1:2:1) at 700°C for different times. The results show that Ge, CaCr_2_O_4_, and Ca_2_GeO_4_ appeared in the cathode after 0.5 h of electrolysis. The generation of CaCr_2_O_4_ and Ca_2_GeO_4_ is caused by combination reactions between Cr_2_O_3_, GeO_2_, Ca^2+^, and O^2−^, which has been confirmed in previous works (Rong et al., 2014; Pang et al., 2018). CaCr_2_O_4_ and Ca_2_GeO_4_ as intermediate phases can also be electrochemically reduced to the corresponding metals of Cr and Ge. With the extension of electrolysis time to 2 h, the target Cr_2_GeC MAX phase accompanied by a portion of Ge, CaCr_2_O_4_, Cr_3_C_2_, and Cr_7_C_3_ was detected. This result also indicates that the reduction of GeO_2_ and/or Ca_2_GeO_4_ is faster compared to that of CaCr_2_O_4_. Furthermore, the reaction between Cr and C is also thermodynamically advantageous, leading to the formation of Cr_3_C_2_ and Cr_7_C_3_. It should be noted that no significant characteristic peak of C was detected due to the use of amorphous structured graphite powder. The final product after electrolysis for 3 h was detected to be the Cr_2_GeC MAX phase. In addition, some weak Cr_3_C_2_ and Cr_7_C_3_ characteristic peaks are still present in the XRD pattern due to the loss of Ge during electrolysis. It is inferred that the loss of the Ge content comes from the electrochemically reduced Ge and the intermediate product of Ca_2_GeO_4_. The former (Ge) in the nano state usually has a low melting point to enter the molten salt, and the latter (Ca_2_GeO_4_) has a certain solubility in molten salts (Wu and Yang, 2001; Zou et al., 2020). Therefore, excess GeO_2_ is necessary for molten salt electrosynthesis of the Cr_2_GeC MAX phase from Cr_2_O_3_/GeO_2_/C.

**FIGURE 3:**
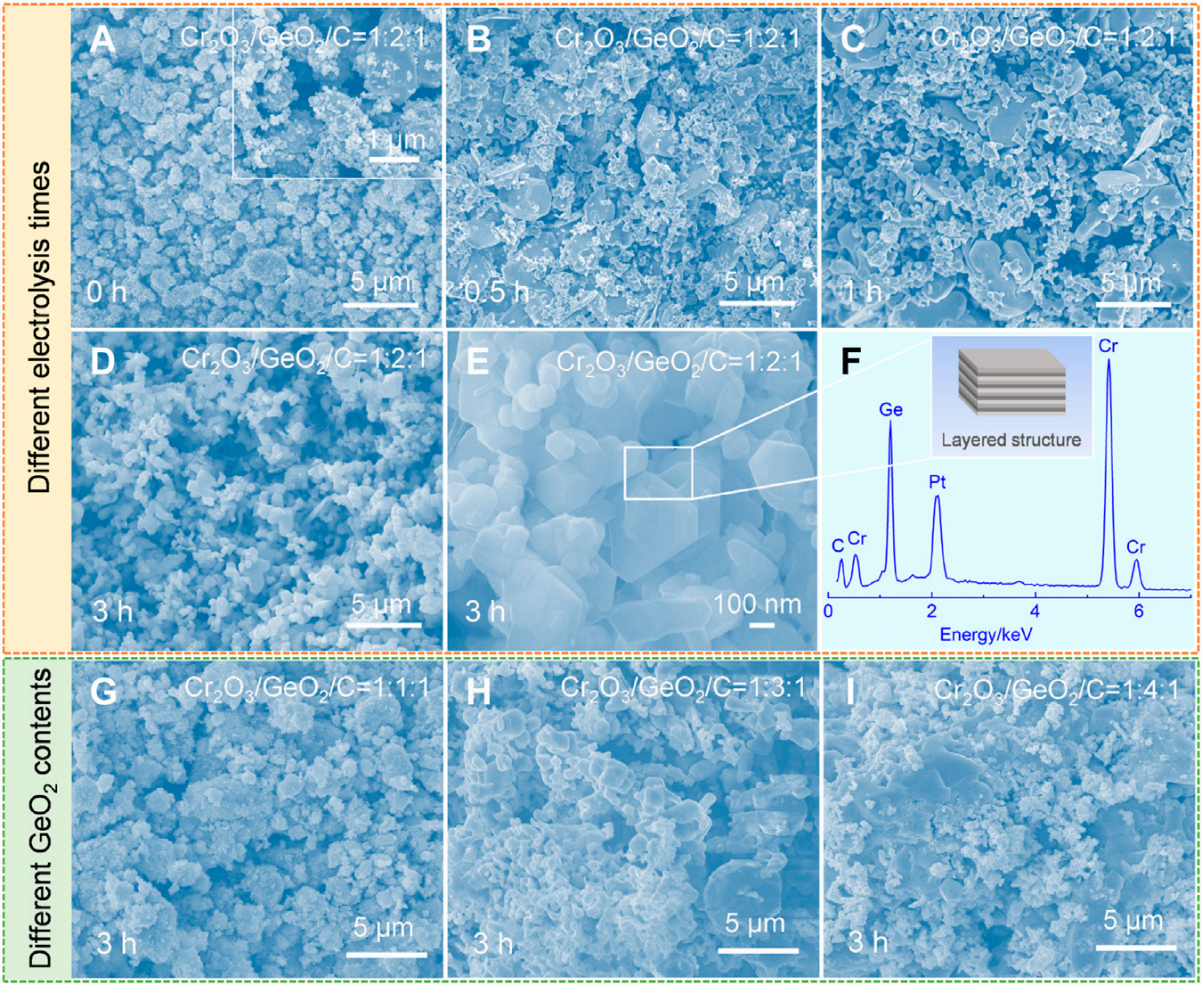
**(A–F)** SEM images and EDS spectra of the samples obtained by the electrolysis of Cr_2_O_3_/GeO_2_/C with a molar ratio of 1:2:1 at 700°C for different times. **(A)** 0 h (i.e., raw material). **(B)** 0.5 h. **(C)** 1 h. **(D,E)** 3 h. **(F)** EDS spectra corresponding to **(E)**. **(G,I)** SEM images of the samples obtained by the electrolysis of Cr_2_O_3_/GeO_2_/C with different molar ratios: **(G)** 1:1:1, **(H)** 1:3:1, and **(I)** 1:4:1.

As the synthesis conditions of Cr_2_GeC have been confirmed, the morphological variation of cathodic samples obtained at different electrolysis conditions was further investigated by SEM. Figure 3A shows the SEM image of the raw Cr_2_O_3_/GeO_2_/C precursor, illustrating a mixture of particles with inhomogeneous size morphologies. The larger particles are Cr_2_O_3_ with a size of about 3 μm, while the nanoparticles are GeO_2_ and C. After 0.5 h of electrolysis (Figure 3B), irregular particles up to 5 μm in size and tiny nodular nanoparticles appeared in the cathode sample, which corresponds to Cr_2_O_3_, CaCr_2_O_4_, Ca_2_GeO_4_, and Ge based on the XRD results. The nodular nanoparticles in the field of view became more numerous with the extension of the electrolysis time to 1 h, as shown in Figure 3C. After 3 h of electrolysis, the obtained sample is the Cr_2_GeC MAX phase, showing a uniform morphology of nanoparticles (Figure 3D). The magnified SEM image (Figure 3E) shows that the as-prepared Cr_2_GeC has a clear layered structure. The apparent characteristic peaks of Cr, Ge, and C were detected by EDS, as shown in Figure 3F. The SEM images of the samples obtained by the electrolysis of Cr_2_O_3_/GeO_2_/C with different molar ratios at 700°C for 3 h are shown in Figures 3G–I. In the case of insufficient GeO_2_ addition (i.e., Cr_2_O_3_/GeO_2_/C with a molar ratio of 1:1:1), the products are Cr_2_GeC, Cr_7_C_3_, and Cr_3_C_2_ mixtures, thus showing a mixed irregular sintered morphology (Figure 3G). However, with the increase of the GeO_2_ content (i.e., Cr_2_O_3_/GeO_2_/C with molar ratios of 1:3:1 and 1:4:1), sintered clusters appeared in the electrolytic samples due to the generation and growth of Ge from excessive GeO_2_, and the increase in the Ge content enables this phenomenon to become more apparent, as shown in Figures 3H, I.

**FIGURE 4:**
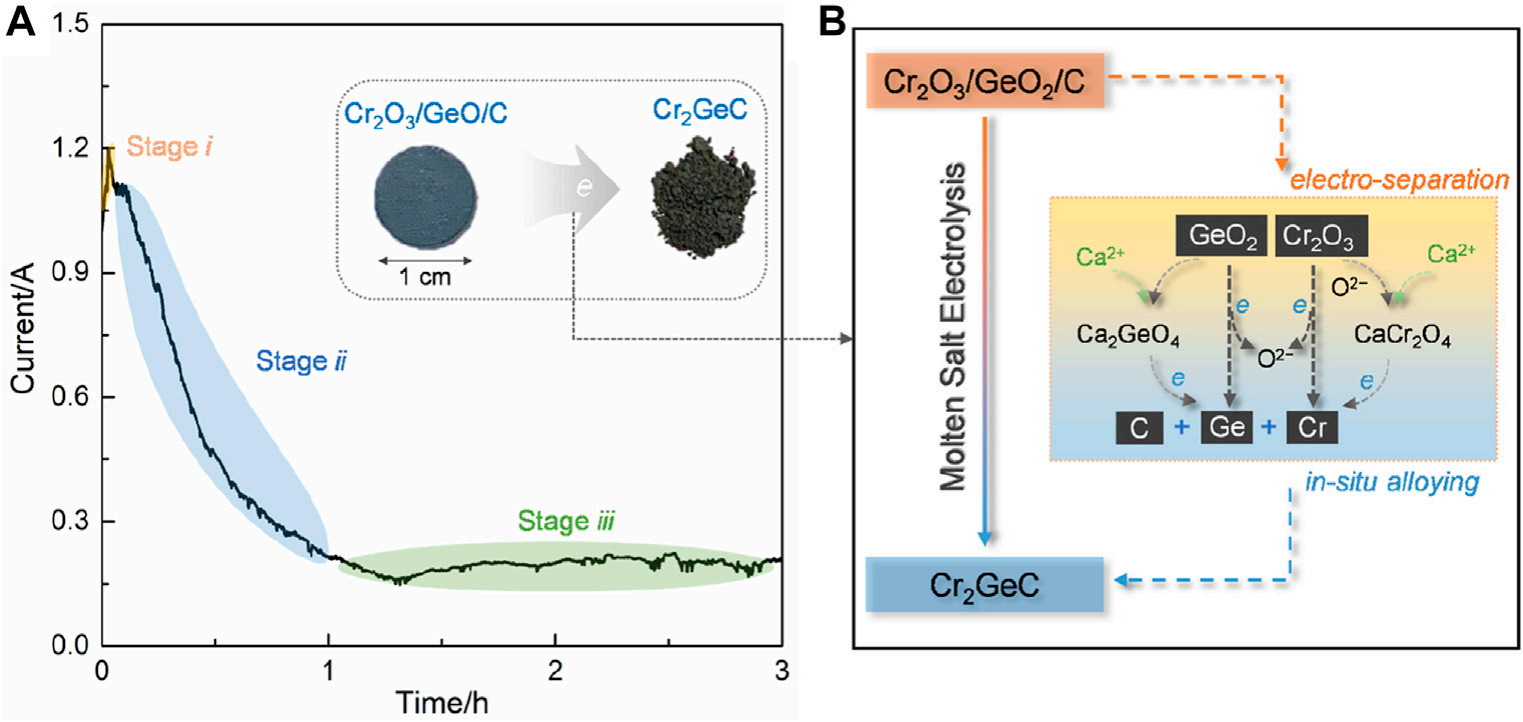
**(A)** Typical current–time curve recorded during the electrolysis of Cr_2_O_3_/GeO_2_/C under 3.0 V at 700°C; the insets are the photographs of the Cr_2_O_3_/GeO_2_/C disc and obtained Cr_2_GeC powders. **(B)** Schematic of the electrochemical synthesis mechanism of Cr_2_GeC from Cr_2_O_3_/GeO_2_/C.

Figure 4A shows the typical current–time curve of the electrolysis of Cr_2_O_3_/GeO_2_/C at 3.0 V. The current value rose to 1.2 A at the brief initial electrolysis stage *i* and then, gradually decreased to about 0.2 A within 1 h (stage *ii*). Subsequently, electrolysis remained in a relatively stable current state until the end of electrolysis (stage *iii*). The current variation during electrolysis can be explained by the three-phase interlines (3PIs) mechanism (Xiao et al., 2006; Xiao and Wang, 2014). The increase of the active surface area coming from the electrochemical reduction of GeO_2_ results in a sharp increase in current. As the 3PIs propagate into the interior of the cathode, the electrolysis process is controlled by oxygen-ion diffusion in the molten salts contained in the cathodic pores, resulting in a decrease in the current. The subsequent stable current value is obtained due to the exhaustion of oxygen in the cathode. The current efficiency (*η*) of this electrolysis process was calculated to be about 38.4% according to the following equation (Ge et al., 2015). This was expected to further improve the current efficiency by optimizing electrolysis systems:

$$\eta =\frac{nFM}{CM}\times 100\%$$ (1)

where n is the number of transferred electrons, F is the Faraday constant, m is the metal mass obtained by electrolysis, C is the charge passed during electrolysis, and M is the relative atomic mass of Cr_2_O_3_ and GeO_2_.

The electrochemical synthesis mechanism of the Cr_2_GeC MAX phase from Cr_2_O_3_/GeO_2_/C was proposed based on the aforementioned analysis, and the corresponding schematic is shown in Figure 4B. GeO_2_ in the cathode was preferentially reduced to metal Ge by reaction (2), as confirmed by the XRD results (Figure 2). In addition, Ca_2_GeO_4_ and CaCr_2_O_4_ as intermediate products were generated through combination reactions between Ca^2+^, O^2−^, GeO_2_, and Cr_2_O_3_ by reactions (3)–(4) during electrolysis, wherein O^2−^ is derived from the electrolysis of the oxides (GeO_2_ and Cr_2_O_3_) and the residual O^2−^ in the molten salts. These oxides were also successively electrochemically reduced to metals Cr and Ge through reactions (2) and (5)–(7). As a result, the as-generated Cr and Ge can *in situ* react with C to form Cr_2_GeC by reaction (8). In general, the electrosynthesis of the Cr_2_GeC MAX phase involves the electro-separation of oxides and *in situ* alloying of Cr, Ge, and C.

$$GeO_{2}+{4e}^{-}=Ge+{2O}^{2-}$$ (2)

$$GeO_{2}+{2Ca}^{2+}+{2O}^{2-}={Ca}_{2}GeO_{4}$$ (3)

$${Cr}_{2}O_{3}+{Ca}^{2+}+O^{2-}=Ca{Cr}_{2}O_{4}$$ (4)

$${Ca}_{2}{GeO}_{4}+{4e}^{-}=Ge+{2Ca}^{2+}+{4O}^{2-}$$ (5)

$${Cr}_{2}O_{3}+{6e}^{-}=2Cr+{3O}^{2-}$$ (6)

$$Ca{Cr}_{2}O_{4}+{6e}^{-}=2Cr+{Ca}^{2+}+{4O}^{2-}$$ (7)

$$2Cr+Ge+C={Cr}_{2}GeC$$ (8)

**FIGURE 5:**
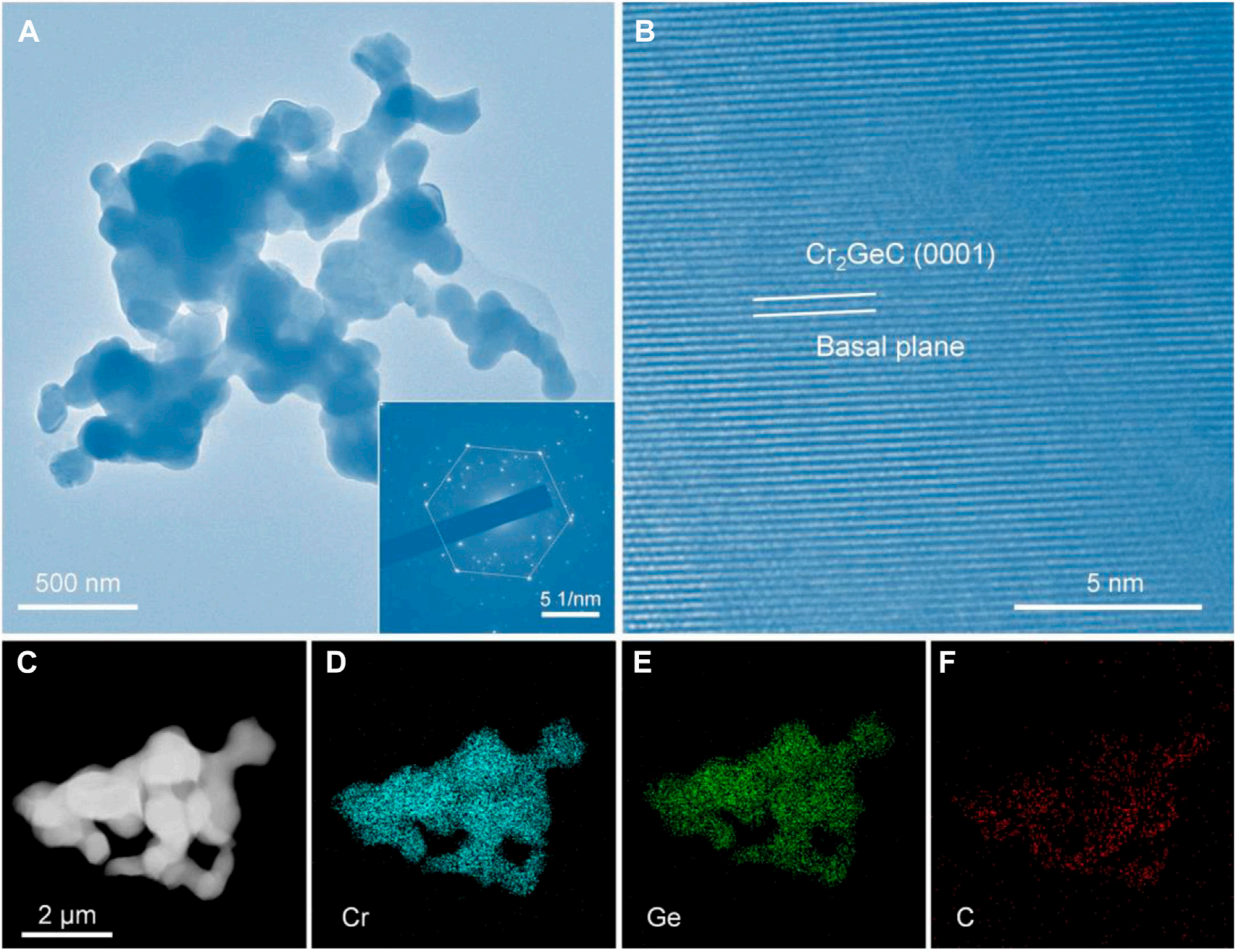
**(A)** TEM and **(B)** high-resolution TEM images of the as-prepared Cr_2_GeC; the inset in **(A)** is its corresponding selected area electron diffraction (SAED) pattern. **(C)** TEM and **(D–F)** are the corresponding EDS mappings of Cr_2_GeC nanoparticles.

The microstructure of the synthesized Cr_2_GeC MAX phase was further investigated by TEM. Figure 5A distinctly shows the TEM image of the as-prepared Cr_2_GeC powder and the inset is its corresponding selected area electron diffraction (SAED) pattern. Evidently, Cr_2_GeC exhibits a nanoscale irregular shape with a particle size of about 100 nm. In addition, Cr_2_GeC nanoparticles show an interconnected morphology because of the sintering effect during the molten salt electrolysis process. The SAED pattern reveals the typical hexagonal property of the Cr_2_GeC MAX phase. From the high-resolution TEM image shown in Figure 5B, the as-prepared Cr_2_GeC reveals the evident layers along the (0001) crystallographic direction. The regular lattice-resolved image commonly confirms periodic crystal structures (Zhao et al., 2022). An EDS analysis was further performed to investigate the element distribution of Cr_2_GeC nanoparticles. The obtained EDS mapping results are shown in Figures 5C–F. It can be seen that elements Cr, Ge, and C show a uniform distribution and have a good overlap with the particles shown in the TEM image (Figure 5C), demonstrating the homogeneity of the as-prepared Cr_2_GeC nanoparticles.

**FIGURE 6:**
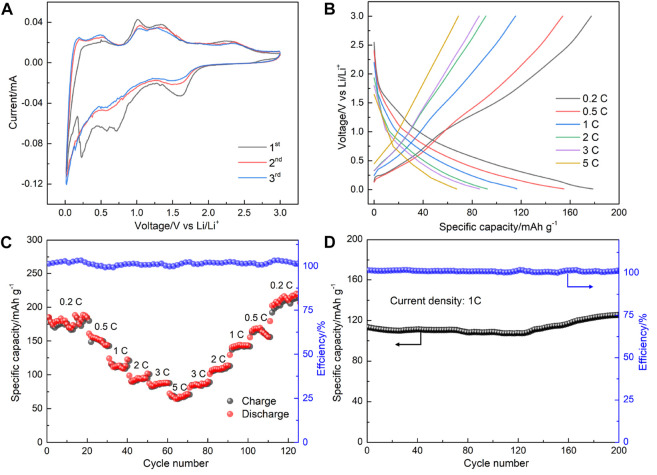
**(A)** Cyclic voltammetry curves at the first, second, and third cycle at 0.1 mV s^−1^ in the potential range of 0.01–3.0 V. **(B)** Galvanostatic charge–discharge curves of the fabricated cell at the current densities range of 0.2–5 C. **(C)** Capacities and Coulombic efficiency at different current densities. **(D)** Cycling performance of the Cr_2_GeC electrode at 1°C for 200 cycles.

To evaluate the lithium-storage performance of the as-prepared Gr_2_GeC MAX phase, Gr_2_GeC nanoparticles were used as anode materials to assemble lithium coin cell batteries for electrochemical tests. Figure 6A shows cyclic voltammetry curves in terms of lithium storage for the first three cycles in the potential range of 0.01–3.0 V with a sweep rate of 0.1 mV s^−1^. It can be seen that several reduction peaks within 0.01–1.0 V appeared only in the first cycle, which may be due to the formation of an irreversible solid electrolyte interface (SEI) phase or the incompletely reversible intercalation of Li in the MAX phase structure (Ren et al., 2016; Xu et al., 2016). The charge–discharge curves and the rate performance of the as-prepared Gr_2_GeC nanoparticles at various current densities are shown in Figures 6B and C. The discharge capacities of Cr_2_GeC are 177.4, 153.5, 112.4, 94.4, 85.7, and 67.6 mAh g^−1^ at current densities of 0.2, 0.5, 1, 2, 3, and 5 C, respectively. The Gr_2_GeC MAX phase exhibits excellent rate capacities for Li-storage. The discharge capacity can recover up to 220.1 mAh g^−1^ upon the reduction of the current rate to 0.2 C, which is superior to the initial discharge capacity of 177.4 mAh g^−1^. In addition, the as-prepared Gr_2_GeC was cycled at a high current density of 1 C for 200 cycles to investigate the cycling performance of Cr_2_GeC. As shown in Figure 6D, the initial discharge capacity of the Cr_2_GeC electrode is 116.4 mAh g^−1^, which increased to 129.8 mAh g^−1^ after 200 cycles and the capacity remained at about 100%. The decreasing size of Gr_2_GeC MAX phase particles and the expansion of two-dimensional structures that are caused by the Ge–Li (de) alloying reaction during (de) lithiation are believed to be responsible for the increase of the capacity during cycling (Li et al., 2021). The aforementioned results visually reveal that the as-prepared Cr_2_GeC nanoparticle can facilitate fast and stable lithium storage. Table 1 shows the comparison of the lithium-storage performance of the synthesized Cr_2_GeC and other reported MAX phase materials (Xu et al., 2016; Chen et al., 2018; Luan et al., 2019; Zhao et al., 2019; Li et al., 2021). It can be seen that the as-prepared Cr_2_GeC also presents promising lithium-storage performance. The energy storage properties exhibited by MAX phase materials are exciting, and molten salt electrolysis provides a facile and controllable strategy for the synthesis of the MAX phase toward energy storage applications.

**TABLE 1:** Comparison of the lithium-storage performance of synthesized Cr_2_GeC and other reported MAX phase materials.

Sample	Capacity (mAh g^−1^)	Current density (C)	References
Gr_2_GeC	177.4	0.2	This work
	116.4	1	
	67.6	5	
V_2_SnC	490	0.5	Li et al. (2021)
	95	50	
Ti_3_SiC_2_	80	4	Xu et al. (2016)
Ti_3_C_2_T_*x*_/Ti_3_AlC_2_	160	1	Chen et al. (2018)
O-doped Ti_3_SiC_2_	230	0.5	Luan et al. (2019)
	85	20	
Nb_2_SnC	115	0.5	Zhao et al. (2019)
	87	5	

**FIGURE 7:**
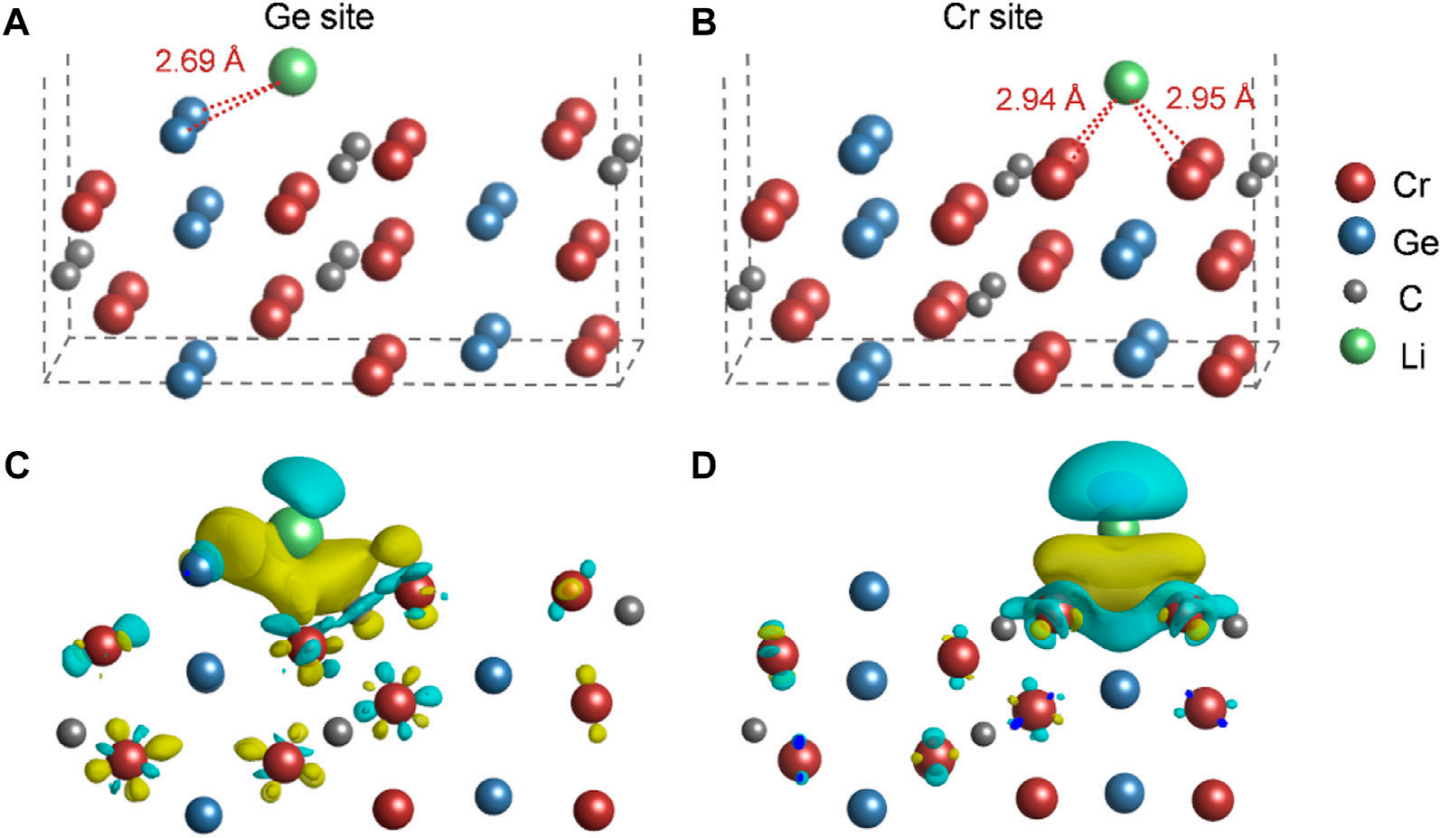
Li ion adsorption in **(A)** Ge and **(B)** Cr sites. The charge redistributions due to the interaction between Li and **(C)** Ge and **(D)** Cr sites. The yellow/blue color represents the charge accumulation/depletion, where isosurfaces refer to an isovalue of 2 × 10^−3^ electrons/Bohr^−3^.

The DFT calculations were performed to preliminarily understand the Li-storage mechanism of Cr_2_GeC. The interaction between Li ions and Cr_2_GeC surfaces was investigated to evaluate the Li-storage behavior of Cr_2_GeC at the atomic scale. As shown in Figures 7A and B, different adsorption sites for Li adsorption were investigated, leading to two stable adsorption configurations. The adsorption energies for Ge and Cr sites are −1.34 eV and −0.69 eV, respectively. The results indicate that the reaction between the Ge atom and the Li ion is more favored. In addition, Bader charge analysis and charge redistribution were performed to further gain a deeper understanding of their adsorption behaviors. The change of the Bader charge in surface elements shows the interaction between the Cr_2_GeC and Li cation. Ge gains 0.019|e| when the Li cation is adsorbed on this site, indicating that Ge–Li reactions are preferred, which occur and contribute to the redox capacity. The side view of charge redistribution for the same isosurface value (2 × 10^−3^ electrons/bohr^−3^) is shown in Figures 7C and D. The yellow/blue color represents the charge accumulation/depletion, suggesting a strong interaction between Li and Ge, which is in accordance to the adsorption energies. The DFT calculation result reveals that Li storage may be caused by Li–Ge alloying at the edges of Cr_2_GeC nanoparticles.

## 4 Conclusion

The Cr_2_GeC MAX phase with a typical two-dimensional layered structure has been electrochemically synthesized in molten salts. This electrosynthesis process consumes only electrons to directly convert Cr_2_O_3_/GeO_2_/C into Cr_2_GeC at a moderate temperature of 700°C. The synthesis mechanism mainly involves the electro-separation of oxygen ions from Cr_2_O_3_/GeO_2_ and *in situ* alloying of the as-generated Cr, Ge, and C. The as-prepared Cr_2_GeC MAX phase shows a uniform morphology of nanoparticles with a particle size of about 100 nm. Cr_2_GeC nanoparticles have been further investigated as anode materials for lithium-ion batteries, which showed attractive electrochemical performance with a specific capacity of 177.4 mAh g^−1^ at 0.2 C and excellent cycling performance. The possible lithium-storage mechanism of Cr_2_GeC has been discussed based on DFT calculations, whereby Ge atoms at edge sites of Cr_2_GeC nanoparticles undergo the (de) alloying reaction of Ge–Li.

## Data Availability

The original contributions presented in the study are included in the article/supplementary material, further inquiries can be directed to the corresponding authors.
